# Themis2 Is Not Required for B Cell Development, Activation, and Antibody Responses

**DOI:** 10.4049/jimmunol.1400943

**Published:** 2014-06-06

**Authors:** Harald Hartweger, Edina Schweighoffer, Sophia Davidson, Matthew J. Peirce, Andreas Wack, Victor L. J. Tybulewicz

**Affiliations:** * Medical Research Council, National Institute for Medical Research, London NW7 1AA, United Kingdom; and; † Kennedy Institute of Rheumatology, Imperial College, London W6 8LH, United Kingdom

## Abstract

Themis1 is a protein implicated in transducing signals from the TCR. Mice deficient in Themis1 show a strong impairment in T cell selection in the thymus and defective T cell activation. The related Themis2 protein is expressed in B cells where it associates with signaling proteins Grb2 and Vav1, and is tyrosine phosphorylated after BCR stimulation. Thus, it has been proposed that Themis2 may transduce BCR signals, and hence play important roles in B cell development and activation. In this article, we show that Themis2 is expressed in all developing subsets of B cells, in mature follicular and marginal zone B cells, and in activated B cells, including germinal center B cells and plasma cells. In contrast, B lineage cells express no other Themis-family genes. Activation of B cells leads to reduced Themis2 expression, although it remains the only Themis-family protein expressed. To analyze the physiological function of Themis2, we generated a Themis2-deficient mouse strain. Surprisingly, we found that Themis2 is not required for B cell development, for activation, or for Ab responses either to model Ags or to influenza viral infection.

## Introduction

Themis-family proteins are defined by the presence of a cysteine-containing, all-β in Themis (CABIT) domain (1). The family can be traced down to cnidarians, but in mammals it is composed of five members: Themis1 (originally Themis, but in this article termed Themis1 for clarity), Themis2, Themis3, Garem, and Gareml. The three Themis proteins share a similar structure including two consecutive CABIT domains and a proline-rich region (PRR), whereas Garem and Gareml contain one CABIT domain, a PRR, and a SAM domain (1–3). Themis1 and Themis2 show high conservation between each other, and both have a putative nuclear localization signal (NLS) and conserved C-terminal tyrosine residues, which serve as SH2-binding sites upon phosphorylation (4–7).

Signals from the BCR and TCR play critical roles in the development, survival, and activation of B and T lymphocytes. Several studies showed that Themis1 is essential for normal T cell development (1, 4, 5, 8, 9). In the absence of Themis1, positive selection of CD4^+^CD8^+^ double-positive thymocytes is impaired. Themis1 is phosphorylated after TCR stimulation, suggesting that Themis1 participates in signaling from the TCR (4, 7, 10–12). A recent study showed that Themis1 dampens signaling in thymocytes after low-affinity TCR stimulation, which normally results in positive selection, but when Themis1 is absent, such low-affinity stimulation results in increased TCR signaling and hence negative selection (13).

In comparison, little is known about Themis2. Studies in cancer cell lines suggested a role for Themis2 in differentiation and proliferation (14). Subsequently, Themis2 was shown to be involved in LPS-induced TNF-α production in RAW cells and primary human macrophages where it interacts with Lyn, Grb2, and Vav1 (6). Themis2 also associates with Grb2 and Vav1 in B cells and is phosphorylated after BCR stimulation (7). Furthermore, expression of Themis2 in the T cell lineage rescues thymocyte development in Themis1-deficient mice, suggesting that Themis1 and Themis2 carry out similar functions (7).

Strikingly, Themis1 and Themis2 show mutually exclusive expression: Themis1 is expressed in T cells, whereas Themis2 is expressed in B cells, macrophages, and dendritic cells (http://www.immgen.org). It has been noted for a long time that there are strong similarities between BCR and TCR signaling pathways (15); often one protein will exert a certain function in T cells, whereas its paralogue performs a similar function in B cells. Given the important role for Themis1 in T cell development and the high degree of similarity between Themis1 and Themis2, we hypothesized that Themis2 may have a critical function in B cell development or activation.

In this article, we show that Themis2 is expressed in all subsets of developing and mature B cells, and that no other Themis-family member is expressed in the B cell lineage. Furthermore, we show that Themis2 expression is downregulated after B cell activation. However, despite this nonredundant expression pattern, we find that, surprisingly, in the absence of Themis2, B cell development, activation, and Ab responses are unaffected.

## Materials and Methods

### Mice

The embryonic stem cell line JM8.F6 with the *Themis2*^tm1a(KOMP)Wtsi^ targeted allele (http://www.komp.org) was used to generate C57BL/6J-*Themis2*^tm1a(KOMP)Wtsi^/Nimr mice. These were crossed to C57BL/6J-Tg(ACTFLPe)9205Dym mice (16) and to C57BL/6J-Tg(Prm-cre)70Og mice (17) to delete the frt-flanked lacZ reporter and the loxP-flanked exon4 of *Themis2* respectively, generating C57BL/6J-*Themis2*^tm1d(KOMP)Wtsi^/Nimr mice (*Themis2*^KO^). All experiments used age- and sex-matched littermate control animals. Other strains used: B6.SJL-Ptprc^a^/Nimr (CD45.1^+^), C57BL/6J-Rag1^tm1Mom^/Nimr (Rag1-deficient), and C57BL/6J-Tg(TcraTcrb)425Cbn/Nimr (OT-II) (18). All mice were bred under specific pathogen-free conditions at the National Institute for Medical Research. Radiation chimeras were generated as described previously (19).

### Immunizations

Mice were injected i.p. with PBS containing 10^9^ SRBC (Innovative Research), 50 μg NP (4-hydroxy-3-nitrophenylacetyl)_(0.6)_-LPS in PBS, 50 μg NP_(40)_-AECM-Ficoll in PBS, 50 μg NP_(21)_-CGG (all BioSearch Technologies) in Alum (Thermo Scientific) with PBS for primary immunizations, or 50 μg NP_(21)_-CGG in PBS for rechallenge and were bled or sacrificed at various time points for analysis or cell sorting. For cholera toxin (CTX) immunization, food was withdrawn from mice 3 h before being orally gavaged first with 1.5% NaHCO_3_ and then with 125 μg/kg CTX from *Vibrio cholerae* Inaba 569B (List Biological Laboratories) in PBS. Blood was withdrawn at the indicated time points. Mice were sacrificed 14 d after immunization to obtain fecal samples from the small intestine.

### Flow cytometry, cell sorting, and cell enrichment

RBC-lysed single-cell suspensions were stained in ice-cold PBS containing LIVE/DEAD fixable near-IR dead cell stain (Life Technologies) and the appropriate pretitered Abs. Cell numbers in the bone marrow are quoted per leg (one femur and one tibia). B10 cells were purified using the Miltenyi Biotec Regulatory B cell isolation kit with 24-h in vitro stimulation followed by flow cytometric sorting for B220^+^CD19^+^IL-10^+^ cells. To isolate plasma cells and plasmablasts, we enriched organ suspensions from mice immunized 5 d earlier with SRBC for CD138^+^ cells by staining with anti–CD138-PE and then using anti-PE beads (Miltenyi Biotec). To isolate germinal center B cells, we depleted splenocytes from mice immunized 10 d earlier with SRBC using anti–CD43-biotin and anti–IgD-biotin followed by streptavidin-Dynabeads (Life Technologies) and then sorted for B220^+^PNA^+^GL7^+^Fas^+^CD38^low^. Data were collected on a BD FACSCanto II or sorted on BD FACSAria II, BD Influx, or Beckman Coulter MoFlo XDP cell sorters. Data were analyzed using FlowJo 9.6 (TreeStar).

### Cell culture

B cells were purified from RBC-lysed single-cell suspensions by magnetic negative depletion using biotinylated Abs against CD43, CD11c, CD11b, and Ly6G and streptavidin-Dynabeads. B cells were cultured in DMEM, 100 μM nonessential amino acids, 20 mM HEPES buffer, 10% FCS, 100 U/ml penicillin, 100 μg/ml streptomycin, 2 mM l-glutamine, 100 μM 2-ME. Unless indicated otherwise, cells were stimulated with 10 μg/ml anti-IgM F(ab)_2_ (Jackson Immunoresearch), 10 μg/ml LPS (Alexis), 1 μg/ml CD40L (R&D Systems), 100 ng/ml IL-4, 100 ng/ml BAFF (both Peprotech), 50 ng/ml PMA, or 500 ng/ml ionomycin (both Sigma-Aldrich).

### Proliferation, survival and cytokine secretion

B cells were isolated and cultured as described above but labeled with 5 μM CFSE in PBS for 10 min at 37°C before culturing for 72 h. Proliferation was assessed by CFSE dilution and number of live cells was measured by a LIVE/DEAD fixable near-IR dead cell stain and counted using PerCP-beads on a flow cytometer. To measure cytokine secretion, B cells were cultured for 48 or 72 h and cytokines in the supernatants were measured with the 16-plex Fluorokine MAP assay (R&D Systems) on a Luminex 100 machine.

### Quantitative RT-PCR

Total RNA was extracted with RNEasy Mini or Micro Plus kits (Qiagen) followed by cDNA synthesis using the Superscript III First Strand Synthesis SuperMix for quantitative RT-PCR (qRT-PCR) kit (Life Technologies). Samples were analyzed on an ABI Fast 7900HT under standard conditions using the TaqMan Gene Expression Assays (Life Technologies). Data were normalized to HRPT1 and analyzed using the comparative threshold cycle method.

### Immunoblotting

Splenic B cells were purified by depletion with biotinylated anti-CD43 Ab and streptavidin-Dynabeads. Cell lysates were prepared using radioimmunoprecipitation assay buffer containing protease inhibitors. Cell debris was removed by centrifugation. Proteins were separated in 7% SDS-PAGE gels and wet-blotted onto Immobilon-FL polyvinylidene difluoride membrane (Millipore) by standard techniques. Membranes were blocked in Odyssey Blocking Buffer (Li-Cor), and then probed with anti-Themis2 exon 4 (sc-160439; Santa Cruz), anti-Themis2 exon 6 (AP9910b; Abgent), or anti–α-tubulin mouse monoclonal (clone TAT-1), followed by appropriate secondary Abs. Signals were detected with an Odyssey Infrared Imager (Li-Cor) and analyzed with the manufacturer’s software.

### RNA sequencing

Follicular B cells were sorted by flow cytometry. Unstimulated samples were lysed directly, and stimulated samples were cultured as described at 3 × 10^6^ cells/ml for 6 h with the indicated stimuli. RNA was isolated using TRIzol and cleaned up using RNeasy mini kit. Single-end, unstranded, poly-A–enriched libraries were made using the TruSeq RNA sample preparation kit (Illumina). Samples were analyzed with an Illumina HiSeq 2000, collecting 13.2–76.1 million reads of 75 bases per sample. Reads were aligned to mm10 (Ensembl version 72) using Tophat (version 2.0.9). Raw counts were determined using the union method in htseq-count (version 0.5.4p3), and mappings were filtered for a phred score >10. edgeR (version 3.2.4) was used for filtration of lowly expressed features, normalization, and statistical analysis. Statistical significance of differences in gene expression was determined using the exact binomial test, reporting differences with a false discovery rate <0.05 (20). For VDJ analysis, reads were mapped to NCBI37/mm10 including an update containing additional VDJ annotation derived from GenBank entries (Avadis NGS). Aligned reads were mapped to the RefSeq transcripts database and normalized by DESeq (21). Gene expression is presented as read density defined as normalized counts per kb of exon. All RNA sequencing (RNAseq) data are deposited in ArrayExpress (https://www.ebi.ac.uk/arrayexpress) with accession number E-MTAB-2499.

### Ag-presentation assay

Splenic B cells were isolated using magnetic negative depletion with PE-conjugated Abs against CD43, CD11b, CD11c, and TCRβ and anti-PE beads. Splenic and lymph node CD4^+^ T cells from OT-II Rag1–deficient mice were isolated using depletion with PE-conjugated Abs against CD11b, CD11c, CD8, and B220. To activate B cells and deliver Ag to the BCR, we stained B cells with 2.5, 0.25, 0.025, or 0 μg/ml anti–IgM F(ab)_2_-biotin and then with OVA Ag delivery reagent (Miltenyi Biotec). A total of 2 × 10^5^ B cells were cocultured with 10^5^ OT-II CD4^+^ T cells in 96-well plates for 72 h. T cell activation was assessed by IL-2 production and measured in the supernatants using the DuoSet mouse IL-2 ELISA (R&D Systems).

### BCR internalization assay

Splenocytes were stained on ice with 10 μg/ml anti-IgM F(ab)_2_-biotin, incubated at 37°C for the indicated times, and BCR internalization stopped with PBS at 0°C. Remaining surface BCR was stained with streptavidin-PE and analyzed by flow cytometry.

### Influenza infection

Mice were infected intranasally with 30 μl PBS containing X31 influenza 50% tissue culture infective dose = 8 × 10^3^ or 8 × 10^4^ and monitored daily for weight loss and clinical symptoms for 15 d and bled at day 10. Mice that reached 75% of their starting weight were euthanized.

### Ig ELISAs

Total serum Ig levels were quantified by ELISA with the SBA Clonotyping System-B6/C57J-HRP (Southern Biotech) according to the manufacturer’s instructions and were revealed with 3,3′,5,5′-tetramethylbenzidine. Signals were quantified using OD at 450 nm of serial dilutions, taking values in the linear portion of the response curve to calculate Ab levels. NP-specific and CTX-specific ELISAs were performed analogously, but plates were coated with 5 μg/ml NP_18_-BSA (BioSearch Technologies) or 1 μg/ml CTX, respectively. For determination of anti-X31 HA-IgG, plates were coated with 2.5 μg/ml of purified X31 HA (John Skehel, National Institute for Medical Research). Serial dilutions of heat-inactivated sera samples were detected with HRP-conjugated goat anti-mouse IgG (Bio-Rad) and 3,3′,5,5′-tetramethylbenzidine.

### Statistical analysis

Statistical analysis was carried out using a Mann–Whitney *U* test.

## Results

### Themis2 is the only Themis-family member expressed in the B cell lineage

**FIGURE 1.**
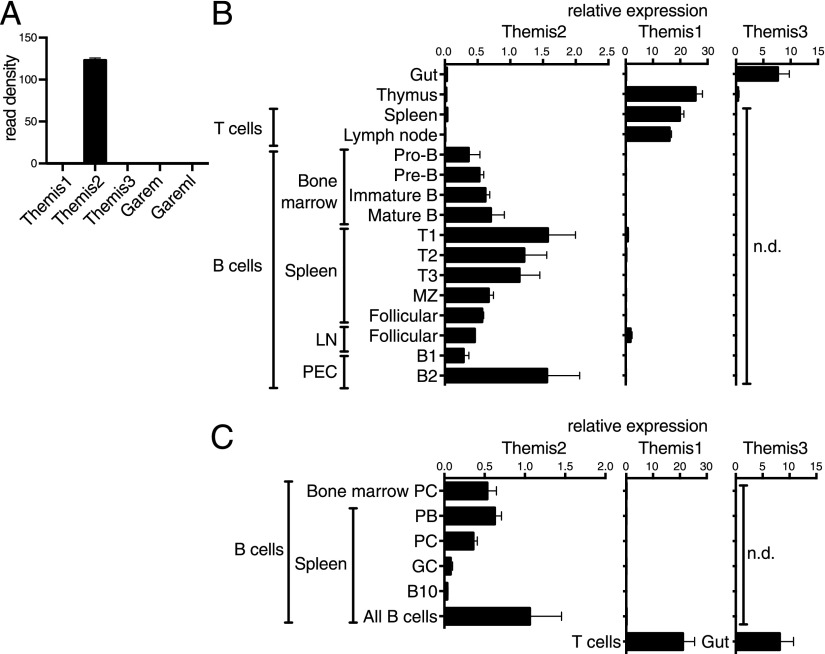
Themis2 is the only Themis-family gene expressed in the B cell lineage. (**A**) Mean (± SEM) mRNA levels of CABIT domain–containing proteins in follicular B cells, measured by RNAseq, displayed as read density. (**B** and **C**) Mean (± SEM) mRNA levels of Themis-family genes in the indicated organs or cell types, measured by qRT-PCR. All cell types were purified by flow cytometric sorting using gating strategies shown in Supplemental Fig. 1. Data are from ≥3 biological replicates per population. GC, germinal center B cell; LN, lymph node; MZ, marginal zone B cell; n.d., not detected; PB, plasmablast; PC, plasma cell; PEC, peritoneal exudate cell.

We first measured expression of Themis-family genes in the B cell lineage. RNAseq analysis of mRNA isolated from mouse splenic follicular B cells showed expression of *Themis2*, but no detectable expression of either of the closely related *Themis1* or *Themis3* genes, or of *Garem* and *Gareml* (Fig. 1A). To extend this analysis, we examined expression of the Themis-family genes in developing subsets of B lineage cells in the bone marrow (pro-B, pre-B, immature B, and mature recirculating B cells) and the spleen (transitional type 1 [T1], T2, and T3 B cells), and in mature B cell subsets in the spleen (follicular and marginal zone B cells), lymph nodes (follicular B cells), and peritoneal cavity (B1 and B2 cells). As a positive control for expression of *Themis1*, we used mRNA isolated from the thymus and from splenic and lymph node T cells, and for *Themis3* we used mRNA from the small intestine. We found that *Themis2* is expressed in all B cell subsets, both developing and mature, with very little or no detectable expression of either *Themis1* or *Themis3* (Fig. 1B). In contrast, thymocytes and T cells express *Themis1*, but not *Themis2* or *Themis3*, and the small intestine expresses exclusively *Themis3*. Finally, we examined expression of Themis-family genes in Ag-activated B cells. Once again we found expression of *Themis2*, but not *Themis1* or *Themis3*, in germinal center B cells, IL-10–secreting B cells (B10 cells), plasmablasts, and plasma cells in the spleen and in plasma cells in the bone marrow (Fig. 1C). However, we noted that expression of *Themis2* was significantly lower in germinal center B cells and B10 cells compared with follicular B cells (*p* = 0.017 and 0.006, respectively). Thus, of the Themis-family, only *Themis2* is expressed in the B cell lineage, with expression detectable in all B cell subsets, although the levels are reduced in Ag-activated B cells.

### Themis2-deficient mice

To study the function of Themis2 in B cells, we generated Themis2-deficient mice on a C57BL/6 background carrying the *Themis2*^tm1d(KOMP)Wtsi^ (*Themis2*^KO^) allele in which exon 4 is deleted. *Themis2*^KO/KO^ mice were viable and produced at expected ratios from heterozygous parents. Exon 4 is the largest exon in the *Themis2* gene and encodes the C terminus of the first CABIT domain, the whole second CABIT domain, NLS, and PRR. Deletion of exon 4 leads to a frameshift between exons 3 and 5, and is thus predicted to eliminate expression of the C terminus of the protein, including Y660, the proposed SH2 domain-binding site.

**FIGURE 2.**
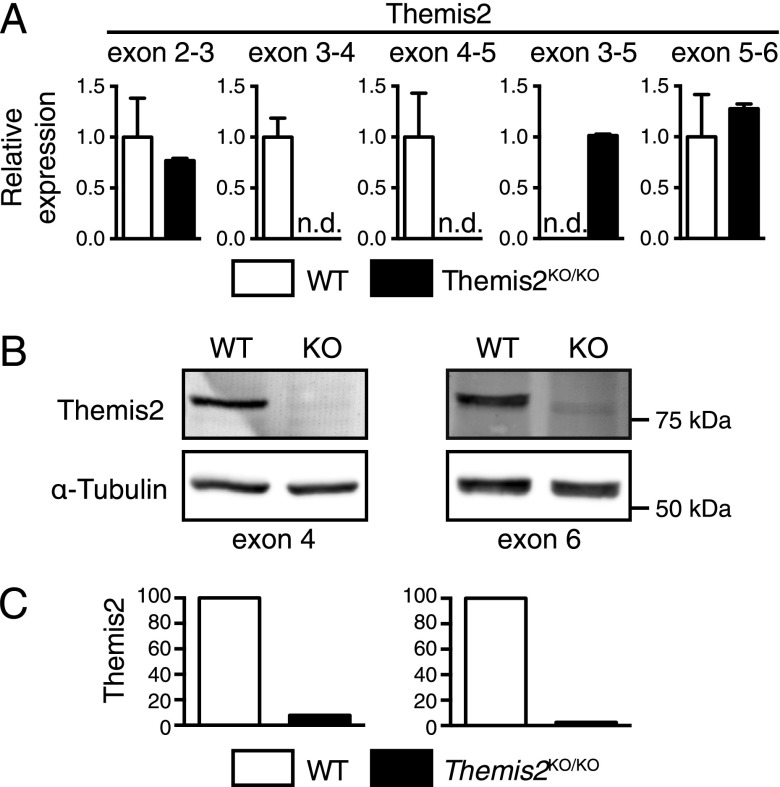
*Themis2*^KO/KO^ mice express a truncated and frame-shifted Themis2 transcript. (**A**) Mean (± SEM) mRNA expression of Themis2 mRNA across the indicated exon junctions determined by qRT-PCR in splenic B cells from WT or *Themis2*^KO/KO^ (KO) mice, normalized to expression in WT cells, which was set to 1. Data are from ≥2 biological replicates. (**B**) Immunoblots of total cell lysates from splenic B cells from WT or KO mice probed with Abs to epitopes of Themis2 encoded in exons 4 or 6, and to α-tubulin. (**C**) Quantification of Themis2 relative to α-tubulin from blots in (B), normalized to expression in WT cells, which was set to 100. Immunoblots are representative of two biological replicates. n.d., not detected.

Analysis of *Themis2* mRNA in splenic B cells from *Themis2*^KO/KO^ mice showed, as expected, no detectable mRNA spanning exons 3–4 and exons 4–5 (Fig. 2A). In contrast, mutant B cells contained normal levels of mRNA spanning exons 2–3 and exons 5–6, and showed splicing from exons 3–5, which was not detectable in wild-type (WT) B cells (Fig. 2A, Supplemental Fig. 2A). We conclude that the effect of the *Themis2*^KO^ mutation is to generate a truncated transcript in which exon 3 is spliced to exon 5. Immunoblot analysis of *Themis2*^KO/KO^ splenic B cell extracts probed with Abs to epitopes in exons 4 and 6 showed no detectable full-length or truncated Themis2 protein (Fig. 2B, 2C). Thus, the mutant allele has eliminated expression of full-length Themis2. However, in the absence of Abs to epitopes in exons 1–3, we cannot exclude that a short N-terminal fragment of Themis2 is still generated, although we note that such a hypothetical protein would contain only part of the first CABIT domain, and no other domain of Themis2.

### Normal B cell development in the absence of Themis2

**FIGURE 3.**
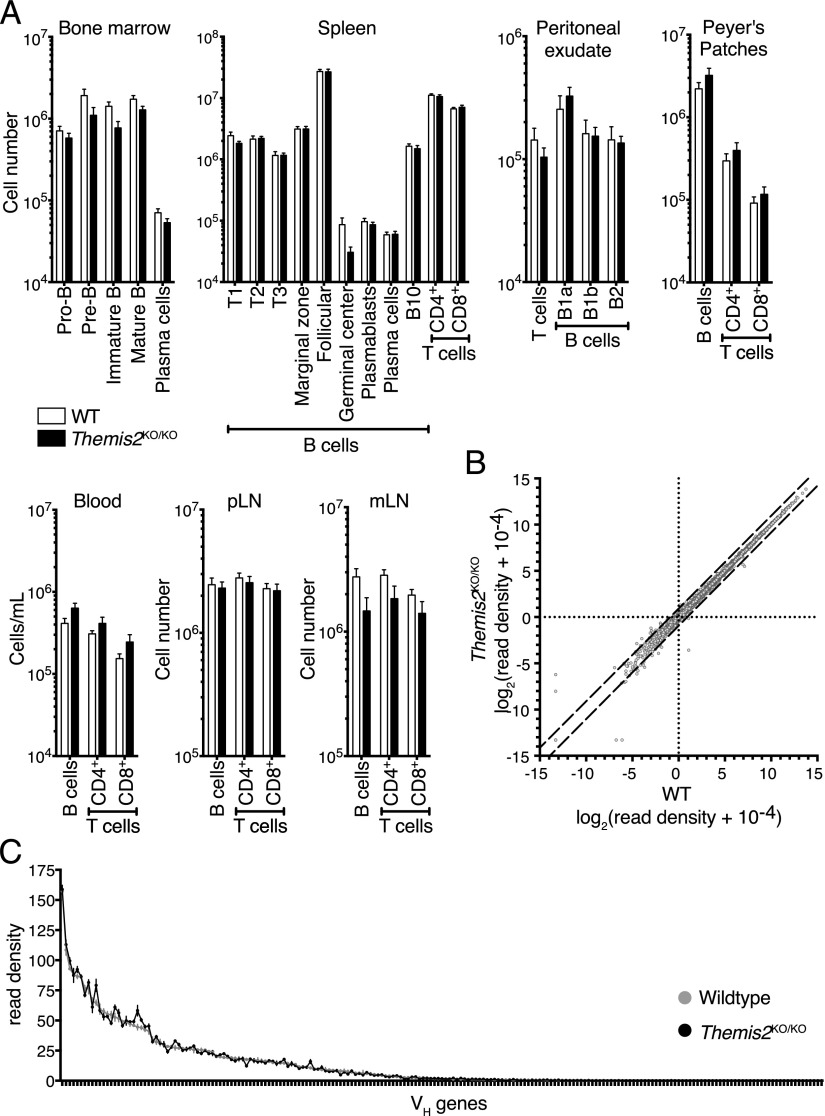
Normal B cell development in *Themis2*^KO/KO^ mice. (**A**) Numbers of B and T cell populations in the indicated organs of *Themis2*^KO/KO^ and WT mice. Cell populations were analyzed using the gating strategy shown in Supplemental Fig. 1. Bars represent mean ± SEM of eight biological replicates. (**B**) Scatterplot showing comparison of gene expression in splenic follicular B cells from *Themis2*^KO/KO^ and WT mice measured by RNAseq and displayed as read density. Each dot is a different gene, dotted lines indicate read density = 1, and dashed lines indicate 2-fold deregulation thresholds. A value of 10^−4^ was added to the read density values so that genes with read density = 0 could be displayed on a logarithmic plot. Data are mean of three biological replicates per genotype. (**C**) Expression of V_H_ genes in splenic follicular B cells from mice of the indicated genotypes as determined by RNAseq. V_H_ genes are ordered by level expression in WT mice. Data are mean (± SEM) of three biological replicates. mLN, mesenteric lymph node; pLN, peripheral lymph node.

To investigate whether Themis2 is required for B cell development, we used flow cytometric analysis to quantitate the numbers of developing and mature B cells in lymphoid tissues of *Themis2*^KO/KO^ mice. We found that the numbers of pro-B, pre-B, immature B, and mature B cells in the bone marrow, and T1, T2, and T3 cells in the spleen were unaltered in the mutant mice (Fig. 3A, Supplemental Fig. 1). Furthermore, there were also no changes in the numbers of mature follicular and marginal zone B cells in the spleen, B1a, B1b, and B2 cells in the peritoneal cavity, and B cells in blood, Peyer’s patches, and peripheral and mesenteric lymph nodes. Finally, we examined Ag-experienced B cells and found that loss of Themis2 did not change the number of plasma cells in the bone marrow, and numbers of germinal center B cells, B10 cells, plasmablasts, and plasma cells in the spleen. We also saw no effect on cell-surface levels of marker proteins on any of these cell types (Supplemental Fig. 1). To extend this analysis, we generated radiation chimeras reconstituted with mixtures of Themis2-deficient and WT bone marrow. Such mixed chimeras provide a competitive environment in which small defects in B cell development are more likely to be apparent. However, these studies once again showed no defect in the ability of Themis2-deficient progenitors to contribute to developing or mature B cell subsets, irrespective of whether the mutant cells made up 50 or 20% of the hematopoietic system (Supplemental Fig. 2B).

To analyze the development of Themis2-deficient B cells further, we used RNAseq to compare genome-wide gene expression between WT and mutant follicular B cells. This analysis showed only three genes that were differentially regulated >2-fold between the two genotypes (Fig. 3B, Supplemental Table 1A). We used these data to also examine usage of variable (V), diversity (D), joining (J), and constant (C) region genes for both heavy (H) and light (κ and λ) Ig chains. Alterations in usage of the V, D, or J region genes could indicate changes in positive or negative selection processes during B cell development. However, we found that usage of V_H_, D_H_, J_H_, V_κ_, J_κ_, V_λ_, and J_λ_ genes was largely identical between WT and Themis2-deficient B cells, as was usage of C_H_, C_κ_, and C_λ_ genes (Fig. 3C, Supplemental Fig. 3). Taken together, these results indicate that B cell development proceeds normally in *Themis2*^KO/KO^ mice.

### Themis2-deficient B cells are activated normally in response to in vitro stimulation

**FIGURE 4.**
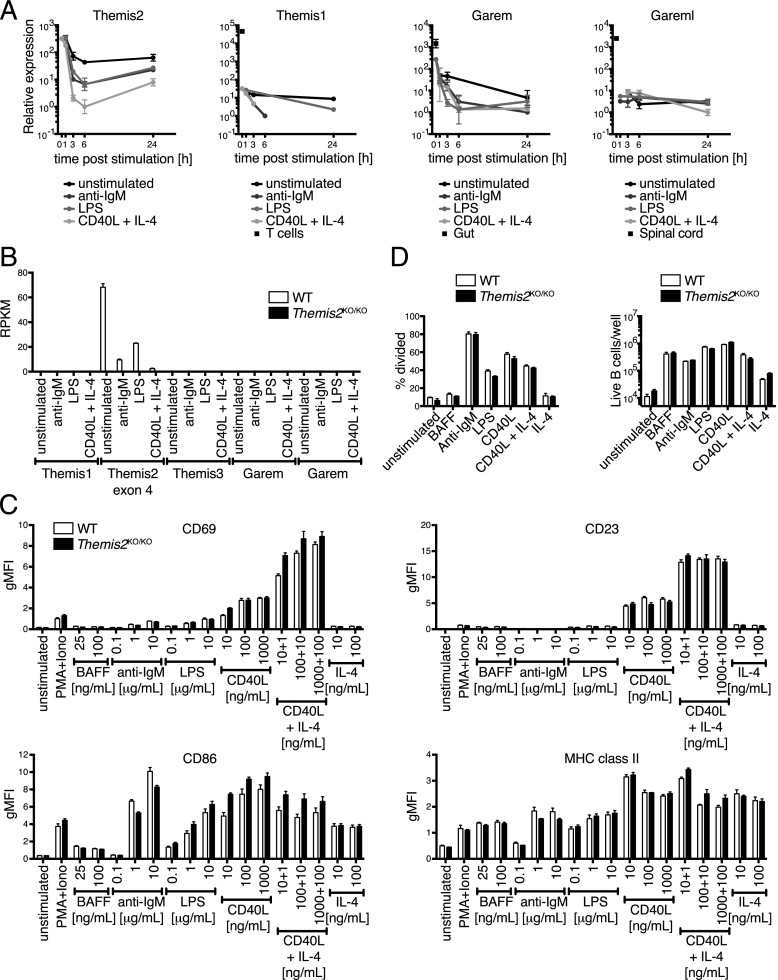
Themis2 is not required for B cell activation, survival, or proliferation. (**A**) Relative mRNA expression data of CABIT domain–containing proteins after in vitro activation of splenic B cells with various stimuli for the indicated time, determined by qRT-PCR. Where data points for Themis1, Garem, or Gareml are missing the transcript could not be detected; Themis3 could not be detected at any time point. Graphs indicate mean ± SEM of three biological replicates and are representative of two independent experiments. (**B**) Expression of mRNA for Themis-family proteins in splenic follicular B cells from WT or *Themis2*^KO/KO^ mice either unstimulated or activated by the indicated stimuli for 6 h. Expression was determined by RNAseq and is shown as mean ± SEM RPKM (reads per kb per million reads) of one to three biological replicates. Expression of Themis1, Themis3, Garem, and Gareml was not detectable. Unstimulated WT data are from the same experiment as in Fig 1A. (**C**) Geometric mean fluorescence intensity (gMFI, arbitrary units) measured by flow cytometry of CD69, CD23, CD86, and MHC class II on splenic B cells in response to various stimuli after in vitro culture for 24 h. Concentrations of stimuli are indicated. Bars indicate mean ± SEM of three biological replicates and are representative of five similar experiments. (**D**) Splenic B cells from WT or *Themis2*^KO/KO^ mice were cultured for 72 h with the indicated stimuli. Graphs indicate mean ± SEM percentage of cells that had divided and numbers of live B cells. Data shown are from three biological replicates and are representative of three similar experiments.

To evaluate a potential role for Themis2 during B cell activation, we first analyzed expression of Themis-family genes in B cells after stimulation in vitro with anti-IgM, LPS, or CD40L and IL-4. We found that the expression of *Themis2* decreased rapidly in response to all three stimuli, reaching a low point after around 6 h, after which the expression increased again (Fig. 4A, 4B). The levels of all other Themis-family genes (*Themis1*, *Themis3*, *Garem*, and *Gareml*) remained very low or undetectable. Furthermore, RNAseq analysis of *Themis2*^KO/KO^ B cells activated by anti-IgM, LPS, or with CD40L and IL-4, showed that no other Themis-family gene was upregulated to compensate for loss of Themis2 (Fig. 4B). Thus, in activated B cells, *Themis2* is the only Themis-family gene expressed, and even in the absence of *Themis2*, no other family member is expressed.

Next, we examined the upregulation of cell-surface markers on WT and *Themis2*^KO/KO^ B cells after activation by anti-IgM, LPS, or with CD40L and IL-4. We found that in response to a range of different doses of these stimuli, the upregulation of CD69, CD23, CD86, and MHC class II was no different in Themis2-deficient B cells compared with WT B cells (Fig. 4C). Furthermore, lack of Themis2 also did not affect either the proliferation or survival of B cells in response to these stimuli (Fig. 4D). BAFFR, a receptor for the cytokine BAFF, transduces signals critical for B cell survival in part via the BCR (19, 22), and thus if Themis2 is involved in BCR signaling, it may also be required for BAFFR signaling. We investigated this possibility by treating WT or Themis2-deficient B cells with BAFF, but found that loss of Themis2 did not affect upregulation of CD86 or MHC class II, or BAFF-induced cell survival (Fig. 4C, 4D).

**FIGURE 5.**
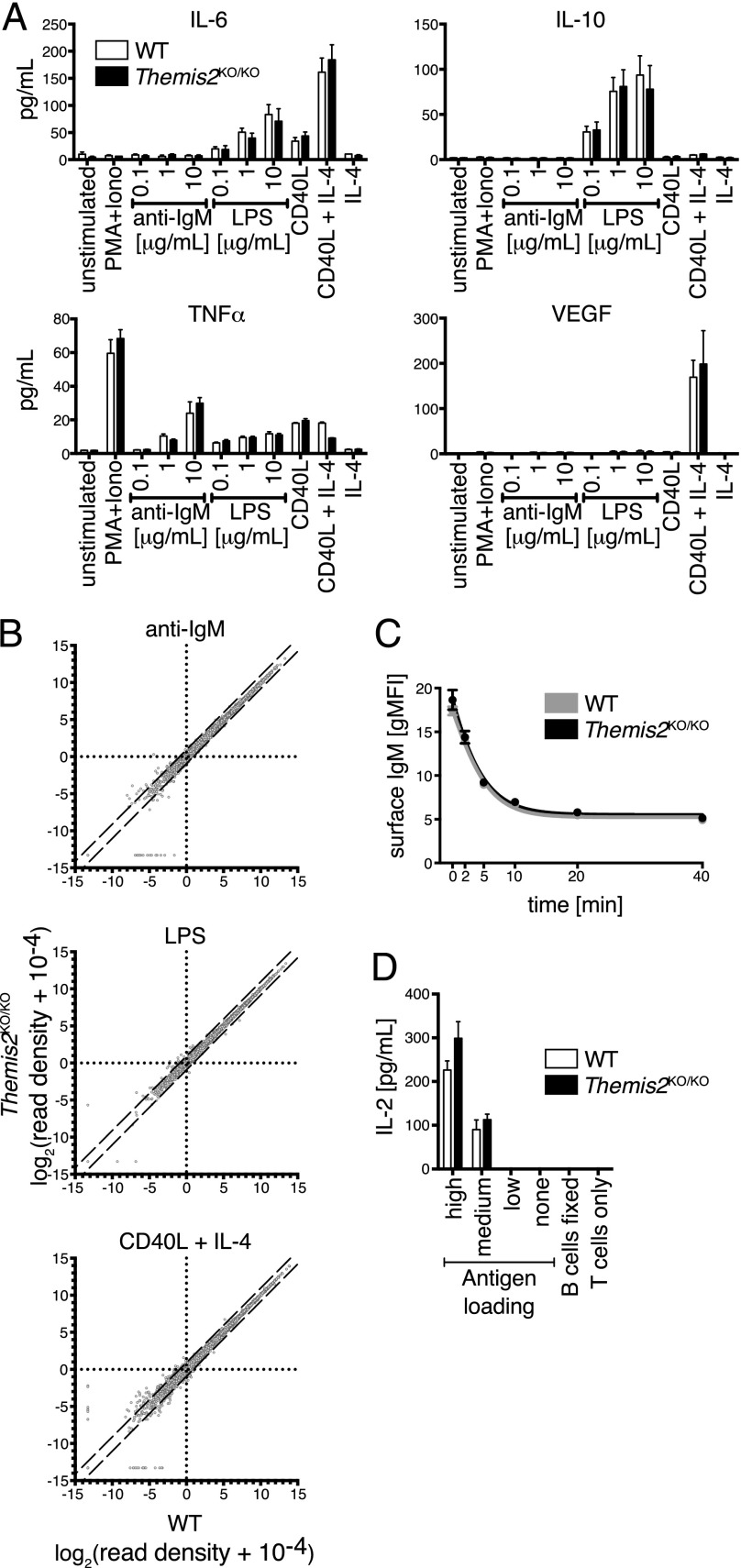
Normal cytokine secretion, gene expression, and Ag presentation by Themis2-deficient B cells. (**A**) Cytokines in the supernatant of B cells cultured for 72 h in the presence of the indicated stimuli. Graphs show mean ± SEM of three biological replicates and are representative of three independent experiments. (**B**) Scatterplots formatted as in Fig. 3B showing comparison of gene expression in splenic B cells from *Themis2*^KO/KO^ and WT mice cultured in the presence of the indicated stimuli for 6 h, measured by RNAseq. (**C**) Surface levels of BCR as a function of time on WT or *Themis2*^KO/KO^ splenic B cells stimulated with anti-IgM F(ab)_2_. Graph shows mean ± SEM of three biological replicates and is representative of three independent experiments. (**D**) IL-2 secretion by OT-II T cells cocultured for 72 h with WT or *Themis2*^KO/KO^ splenic B cells that had been preloaded with low, medium, or high doses of OVA Ag, or with no Ag. As a control, OT-II T cells were also either cocultured alone or with B cells that had been given a high dose of OVA Ag and then fixed. Neither of the latter conditions resulted in any detectable IL-2 secretion, confirming that cytokine secretion by the T cells was dependent on Ag internalization and presentation by B cells. No IL-2 was detected in the absence of T cells (data not shown). Graph shows mean ± SEM of two to three biological replicates and is representative of two independent experiments.

To extend this analysis, we measured secretion of 16 different cytokines (CCL2, CXCL1, CXCL2, GM-CSF, IFN-γ, IL-1β, IL-2, IL-4, IL-5, IL-6, IL-10, IL-12, IL-13, IL-17, TNF-α, and VEGF) after in vitro stimulation of B cells with anti-IgM, LPS, or with CD40L and IL-4. Depending on the stimulus, we were able to detect substantial secretion of only four of these cytokines (IL-6, IL-10, TNF-α, and VEGF), but in all cases, loss of Themis2 did not affect the secretion of these proteins (Fig. 5A). RNAseq studies to measure gene expression in mutant or WT B cells stimulated with anti-IgM, LPS, or CD40L and IL-4 showed that in these 3 conditions, only 3, 8, and 17 genes, respectively, were differentially expressed >2-fold between Themis2-deficient and control B cells (Fig. 5B, Supplemental Table 1B–D). In conclusion, *Themis2*^KO/KO^ follicular B cells are transcriptionally very similar to WT B cells, both before and after activation.

Finally, we examined the ability of Themis2-deficient B cells to internalize and present Ag. Stimulation of splenic B cells with anti-IgM to cross-link the BCR led to internalization of the receptor at the same rate in both WT and Themis2-deficient cells (Fig. 5C). To measure the ability of *Themis2*^KO/KO^ B cells to present Ag to T cells, we treated B cells with an anti–IgM-OVA conjugate and then incubated them with OT-II CD4^+^ T cells bearing a monoclonal TCR specific for an OVA peptide (18) and assayed IL-2 secretion from the T cells as a readout of T cell activation. We found that at three different OVA doses, Themis2-deficient B cells were able to present Ag just as efficiently as WT B cells (Fig. 5D). Taken together, these results show that loss of Themis2 does not perturb B cell activation in vitro or their ability to present Ag to T cells.

### Normal Ab responses in Themis2-deficient mice

**FIGURE 6.**
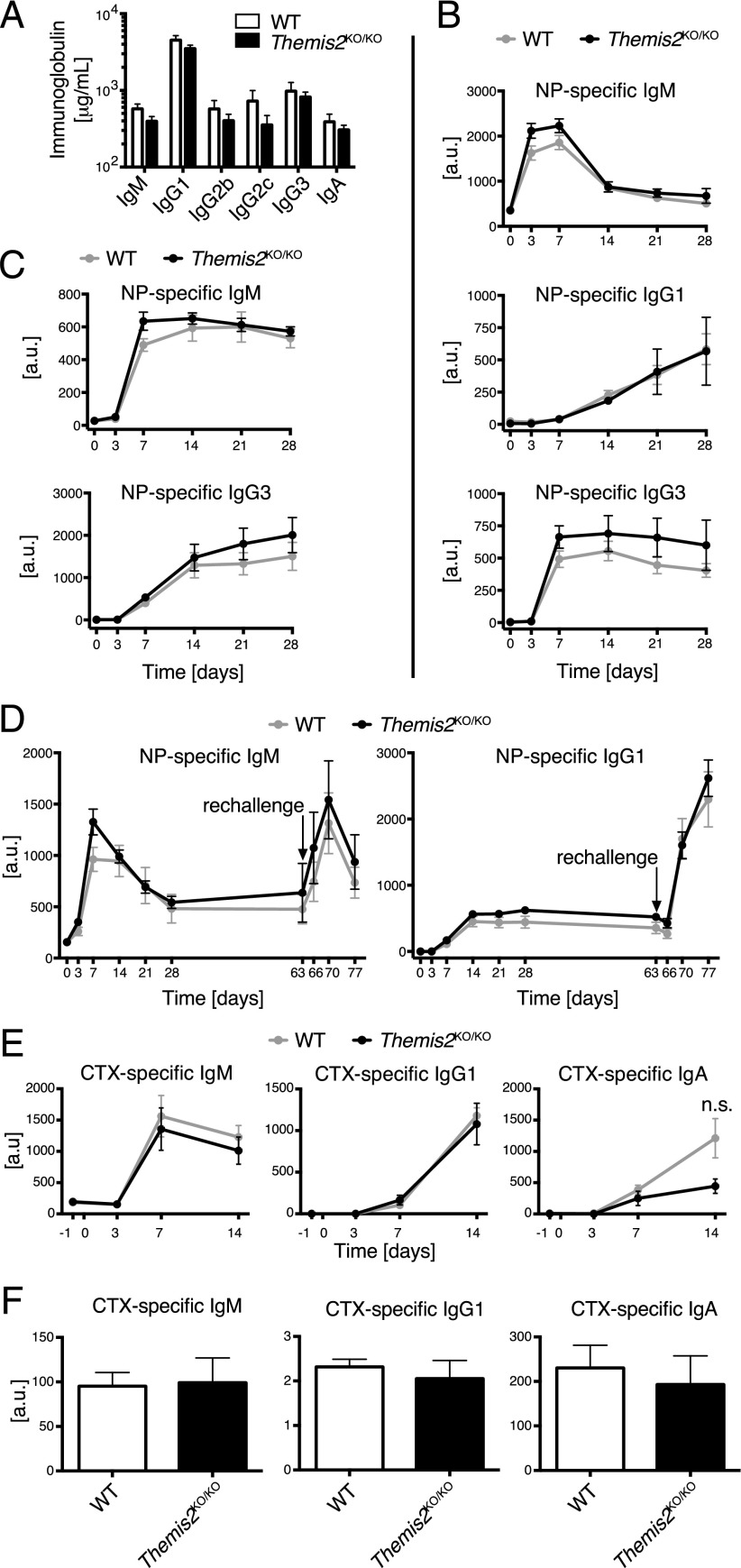
Normal Ab responses in Themis2-deficient mice. (**A**) Serum levels of Ig isotypes in WT and *Themis2*^KO/KO^ mice. Graph shows mean ± SEM of six biological replicates. (**B**–**D**) NP-specific serum Ig levels in WT and *Themis2*^KO/KO^ mice as a function of time after immunization with NP-LPS (B), NP-Ficoll (C), or NP-CGG in alum (D) on day 0. Mice immunized with NP-CGG were rechallenged with NP-CGG in PBS on day 63. Graphs show mean ± SEM of six biological replicates per group. (**E**) CTX-specific serum Ig levels as a function of time or (**F**) fecal Ig levels on day 14 in WT and *Themis2*^KO/KO^ mice after oral CTX immunization. a.u. arbitrary unit; n.s. not significant.

Next, we investigated whether Themis2 was required for in vivo Ab responses. Themis2-deficient mice had normal levels of serum Ig isotypes (Fig. 6A). Immunization of mice with the T-independent type I Ag NP-LPS showed that WT and Themis2-deficient mice induce very similar levels of NP-specific IgM, IgG1, and IgG3 (Fig. 6B). Similarly, the Ab response of Themis2-deficient mice to the T-independent type II Ag NP-Ficoll was also normal (Fig. 6C). Analysis of mice immunized with NP-CGG (chicken γ-globulin) in alum, a T-dependent Ag, showed that *Themis2*^KO/KO^ mice mount a normal anti-NP IgM and IgG1 response (Fig. 6D). The same mice were rechallenged on day 63 with NP-CGG in PBS, and both mutant and control mice showed robust Ag-specific secondary responses (Fig. 6D). Finally, we evaluated the ability of Themis2-deficient mice to mount a mucosal Ab response to orally delivered CTX. Once again, *Themis2*^KO/KO^ mice showed a normal CTX-specific Ab response both in the serum and in feces (Fig. 6E, 6F). Taken together, these data clearly demonstrate that Themis2 is dispensable for Ab responses, at least to model Ags.

### Resistance to influenza infection is unchanged in Themis2-deficient mice

**FIGURE 7.**
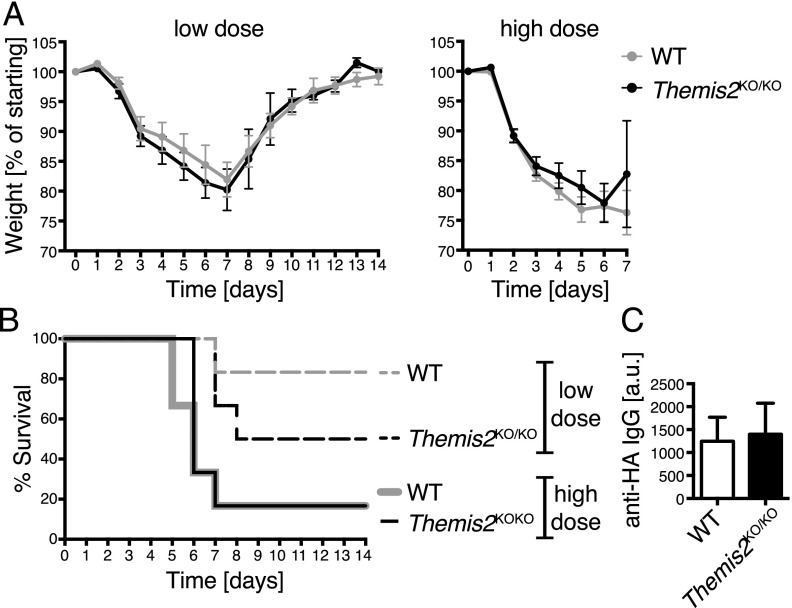
*Themis2*^KO/KO^ mice respond normally to influenza infection. (**A**) Weight of WT or *Themis2*^KO/KO^ mice as a function of time after intranasal infection with X31 influenza 50% tissue culture infective dose = 8 × 10^3^ (low dose) or 8 × 10^4^ (high dose). Data beyond day 7 for the high-dose group is not shown because of high mortality. Graphs show mean ± SEM of six mice per group. (**B**) Kaplan–Meier survival curve of WT or *Themis2*^KO/KO^ mice from the experiment shown in (A). (**C**) Titer of anti-HA IgG in serum from mice 10 d after X31 influenza infection. Bars indicate mean ± SEM of four to six mice per group. a.u., arbitrary unit.

Finally, we assessed the ability of Themis2-deficient mice to mount an immune response to a pathogen. Control and *Themis2*^KO/KO^ mice were challenged intranasally with influenza virus, using either a low dose of virus from which most C57BL/6 mice survive, or a high dose, which is usually lethal. With both doses, Themis2-deficient mice showed weight loss and mortality rates that were similar to WT mice, and both genotypes of mice generated similar levels of serum IgG specific to influenza HA protein (Fig. 7A–C). Thus, Themis2 does not appear to play an important role in the immune response to infection by influenza virus. Because Themis2 is expressed in myeloid cells such as macrophages and dendritic cells, in addition to B cells, and these cell types play important roles in an antiviral immune response (23), we conclude that Themis2 plays no critical role in the antiviral immune response, both in B cells and in myeloid cells.

## Discussion

Previous studies demonstrating a critical role for Themis1 in T cell development and activation, and the reported reciprocal mutually exclusive expression of Themis1 in T cells and Themis2 in B cells, led us to hypothesize that Themis2 may play an analogous important role in B cell development, activation, or function. Such a possible related function was further supported by the report that ectopic expression of Themis2 in the T cell lineage rescues thymocyte development in Themis1-deficient mice (7). We show in this article that the only Themis-family member expressed in B lineage cells is Themis2, with no expression of either Themis1 or Themis3, or of the single CABIT domain proteins Garem and Gareml. Furthermore, we show that this exclusive expression is seen in all subsets of B cells examined, including developing B cells in the marrow, mature B cells in the spleen and lymph nodes, B1 cells in the peritoneal cavity, and in activated B cell subsets. Comparison of Themis2 expression showed that germinal center B cells and B10 cells have the lowest level of expression, suggesting that activation of B cells results in a decrease in Themis2 expression. In agreement with this, we found that activation of B cells in vitro with anti-IgM, LPS, and CD40L+IL-4 results in a rapid decline of Themis2 expression, with no compensating upregulation of expression of any other Themis-family gene. Similarly, previous studies had shown that expression of Themis2 in macrophages is reduced in mice undergoing infection or inflammation (6).

To investigate the function of Themis2, we generated a mouse strain deficient in Themis2 by deleting exon 4 of *Themis2*. The resulting *Themis2* allele is still transcribed, resulting in an mRNA with exon 3 spliced to exon 5, which is predicted to result in a frameshift and subsequent termination of translation in exon 6. As expected, immunoblot analysis of B cell extracts from these mutant mice showed that no full-length or truncated Themis2 protein was detectable using Abs recognizing epitopes in exons 4 and 6. It remains possible that an N-terminal fragment of Themis2 is still made in this strain, although we were unable to test for this in the absence of Abs to epitopes encoded by exons 1, 2, or 3 of *Themis2*. However, such a hypothetical truncated Themis2 protein would include only the N-terminal part of the first CABIT domain of Themis2. It would not contain the rest of this domain, the second CABIT domain, NLS, PRR, or Y660.

It is unlikely that such a truncated protein would be functional for several reasons. First, the PRR and Y660 of Themis2 are required in macrophages for binding of Themis2 to Grb2 and Lyn, respectively (6). Second, mutation of either the PRR or Y660 impairs the ability of Themis2 to promote LPS-induced release of TNF-α. Third, the PRR of Themis1 is required for binding to Grb2 in T cells and for the recruitment of Themis1 to the LAT adapter protein, and hence the immunological synapse (12). Fourth, the CABIT domains, NLS and PRR of Themis1, are required for normal T cell development (11). Finally, a point mutation in the C-terminal end of the second CABIT domain of Themis1 leading to a premature stop codon also abrogates thymocyte development, demonstrating that a Themis1 protein containing only the first CABIT domain is not sufficient to maintain normal function (1). Taken together, these results suggest that if a truncated Themis2 protein is generated from the *Themis2*^KO^ allele, it is unlikely to be functional.

Analysis of *Themis2*^KO/KO^ mice showed that, surprisingly, Themis2 is not required for normal B cell development. No developmental defect was seen even under competitive conditions, where small deficits are more likely to be visible. Analysis of V, D, and J gene usage for both H and L chain Ig genes showed no significant changes in Themis2-deficient mice, indicating that loss of Themis2 did not measurably affect B cell selection. Similarly, loss of Themis2 did not affect in vitro activation of B cells in response to stimulation through the BCR, TLR4, CD40, or IL4R, and did not perturb BCR internalization or Ag presentation to T cells. Finally, Themis2 was not required for Ab responses either to T-independent or T-dependent model Ags, or to influenza virus during the immune response to infection.

This lack of phenotype could potentially be because of functional redundancy between Themis2 and other related proteins. However, RNAseq analysis showed that Themis2-deficient B cells did not upregulate expression of other Themis-family genes. Thus, B lineage cells in *Themis2*^KO/KO^ mice do not express any Themis-family member, making it unlikely that the lack of phenotype in *Themis2*^KO/KO^ mice is due to redundancy with Themis-family proteins. Another possibility for the lack of phenotype is that the *Themis2* gene is nonfunctional. This is unlikely for two reasons. First, the *Themis2* gene is found in all vertebrates, indicating that Themis2 is likely to have an important function (1). Second, it is possible that the *Themis2* gene in laboratory mice has become nonfunctional. However, this is also unlikely because ectopic expression of mouse *Themis2* in the T cell lineage can rescue T cell development in Themis1-deficient mice (7). Taken together, these observations suggest that Themis2 has an important physiological role, but not in the processes examined in this study. We note that Themis2 is expressed in myeloid cells, and it may be that Themis2 is more important in these cells.

In summary, we have shown that Themis2 is expressed in all subsets of developing, mature and activated B cells. Surprisingly, however, loss of Themis2 does not perturb B cell development, activation, or Ab responses. Further studies are needed to identify the physiological function of this conserved protein.

## Supplementary Material

Data Supplement
