# Supplementary material for: Themis2 Is Not Required for B Cell Development, Activation, and Antibody Responses
Source: J Immunol. 2014 Jun 6;193(2):700–7. doi: 10.4049/jimmunol.1400943 (PMC4082722; doi:10.4049/jimmunol.1400943)
Supplement: Data Supplement [file supp_193_2_700__index.html]

Themis2 Is Not Required for B Cell Development, Activation, and Antibody Responses — Themis2 Is Not Required for B Cell Development, Activation, and Antibody Responses — Data Supplement 

# Themis2 Is Not Required for B Cell Development, Activation, and Antibody Responses

## Data Supplement

**Files in this Data Supplement:**

- Supplemental Figures 1 (PDF)
- Supplemental Material 1 (XLSX)
