# Supplementary material for: Themis2 Is Not Required for B Cell Development, Activation, and Antibody Responses
Source: J Immunol. 2014 Jun 6;193(2):700–7. doi: 10.4049/jimmunol.1400943 (PMC4082722; doi:10.4049/jimmunol.1400943)
Supplement: Data Supplement [file 1400943_JI_1400943_Supplemental_Figures_1.pdf]

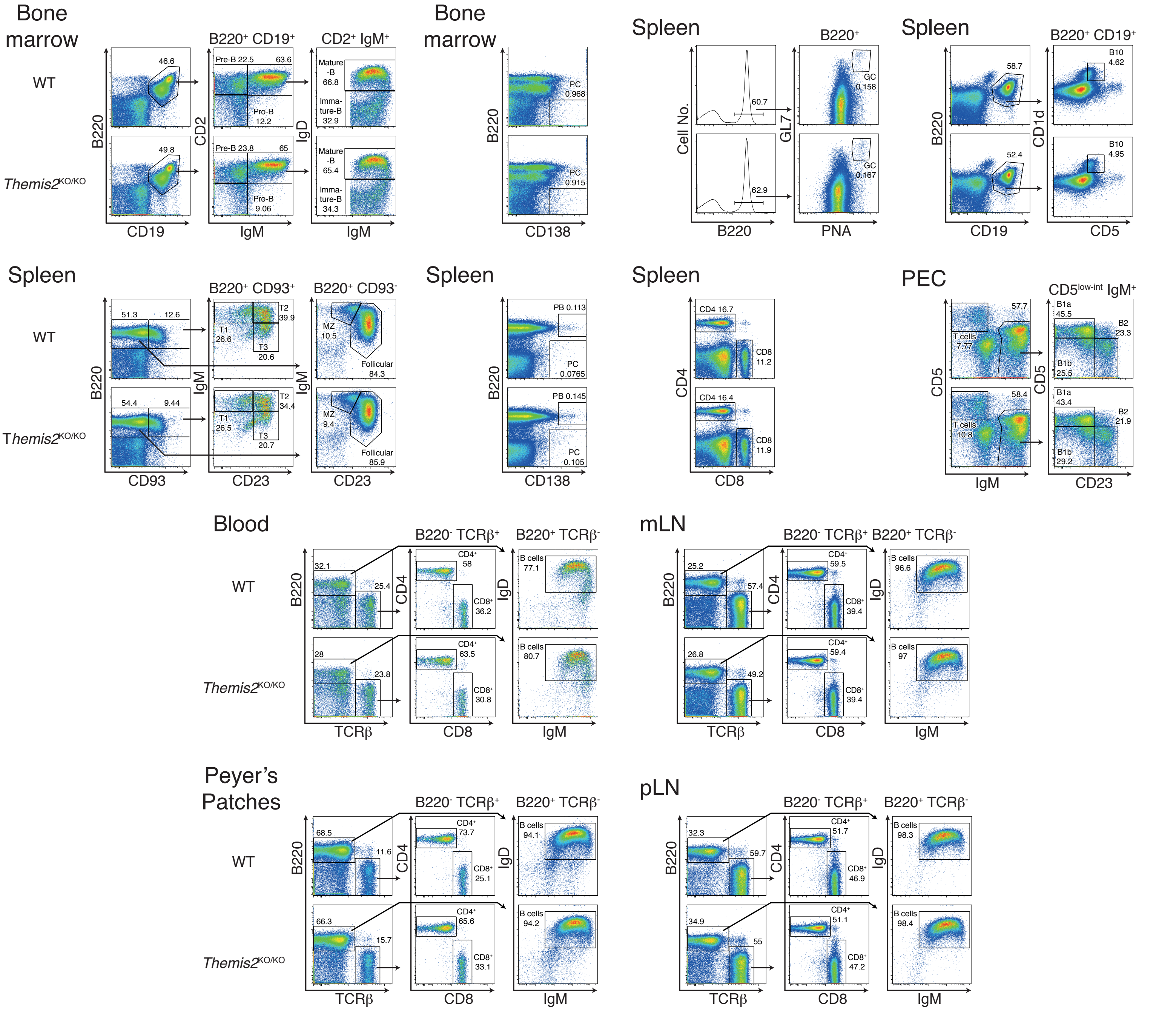

Supplemental Figure 1. Normal B cell development in *Themis2^{KO/KO}* mice. Flow cytometric gating strategies used for sorting cell populations in Fig. 1 and determining cell numbers reported in Fig. 3. Names next to gates indicate the population; numbers beneath indicate the percentage of cells on the total plot falling within the marked gate. Gating strategies were as follows: in bone marrow, pro-B cells (B220+CD19+CD2-IgM-), pre-B cells (B220+CD19+CD2+IgM-), immature B cells (B220+CD19+CD2+IgM+IgD-), mature B cells (B220+CD19+CD2+IgM+IgD+); in spleen, transitional type 1 (T1, B220+CD93+CD23-IgM+), type 2 (T2, B220+CD93+CD23+IgM+) and type 3 (T3, B220+CD93+CD23+IgMlo) B cells, marginal zone B cells (MZ, B220+CD93-CD23-IgM+), follicular B cells (B220+CD93-CD23+IgM+), plasmablasts (PB, B220+CD138+) plasma cells (PC, B220-CD138+), germinal center B cells (GC, B220+PNA+GL7+) and B10 cells (B220+CD19+CD1d+CD5+), CD4 T cells (CD4+), CD8 T cells (CD8+); in peritoneal exudate cells (PEC), B1a cells (IgM+CD5+CD23-), B1b cells (IgM+CD5-CD23-) and B2 cells (IgM+CD5-CD23+), T cells (CD5+IgM-); blood, mesenteric lymph nodes (mLN), peripheral lymph nodes (pLN) and Peyer's patches, B cells (B220+TCRβ-IgM+IgD+), CD4 T cells (B220-TCRβ+CD4+CD8-), CD8 T cells (B220-TCRβ+CD4-CD8+).

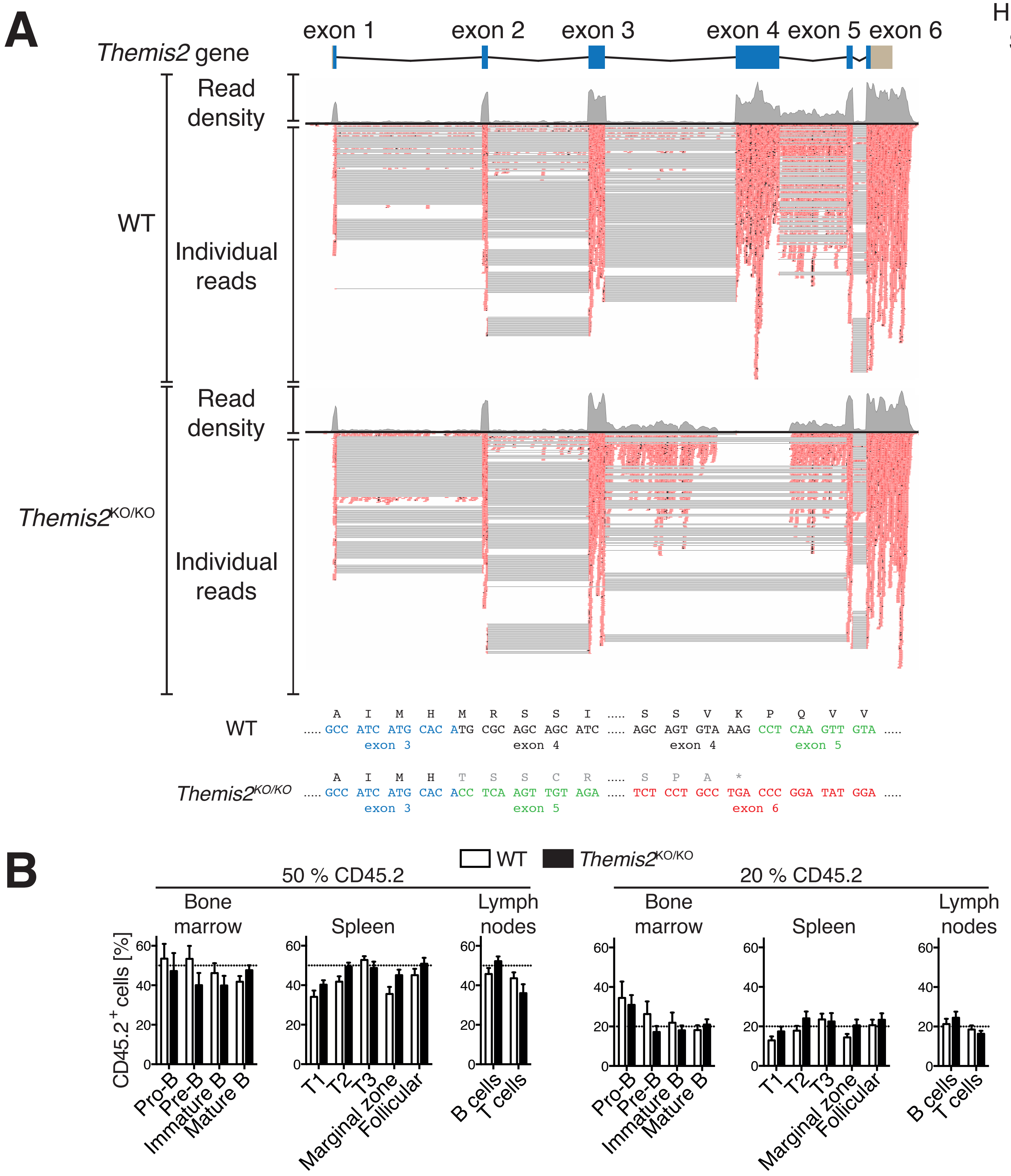

Supplemental Figure 2. *Themis2* mRNA in *Themis2*$^{KO/KO}$ B cells and B cell development in competitive radiation chimeras. (**A**) Alignment of RNAseq data from wild-type (WT) and *Themis2*$^{KO/KO}$ follicular B cells to *Themis2* gene, showing loss of *Themis2* exon 4 and splicing from exon 3 to exon 5 in *Themis2*$^{KO/KO}$ cells. Light brown: UTR; dark blue: coding sequence; grey profile plot: maximum read density; stacked red boxes: individual reads; black lines: connection between two parts of a split read showing splicing. Sequence of Themis2 mRNA derived from RNAseq is shown below, indicating relevant exons and predicted translation. In *Themis2*$^{KO/KO}$ B cells exon 3 is spliced directly to exon 5 resulting in a frameshift in translation (lighter font) and ending in a stop codon (*) in exon 6. (**B**) Irradiated Rag1-deficient mice were reconstituted with bone marrow from CD45.2+ WT or *Themis2*$^{KO/KO}$ mice, mixed with bone marrow from CD45.1+ WT mice, using either 20% or 50% CD45.2+ cells. Graphs show percentage CD45.2+ cells in different cellular fractions in bone marrow, spleen and lymph nodes, from radiation chimeras reconstituted with bone marrow of the indicated genotype at the indicated ratio. Cell numbers in populations were evaluated using flow cytometric gating as in Supplemental Fig. 1 with the addition of staining for CD45.1 and CD45.2. Dotted lines indicates ratio of CD45.2+/CD45.1+ cells in the bone marrow used to reconstitute chimeras. Graphs show mean ± SEM of 6 mice/group.

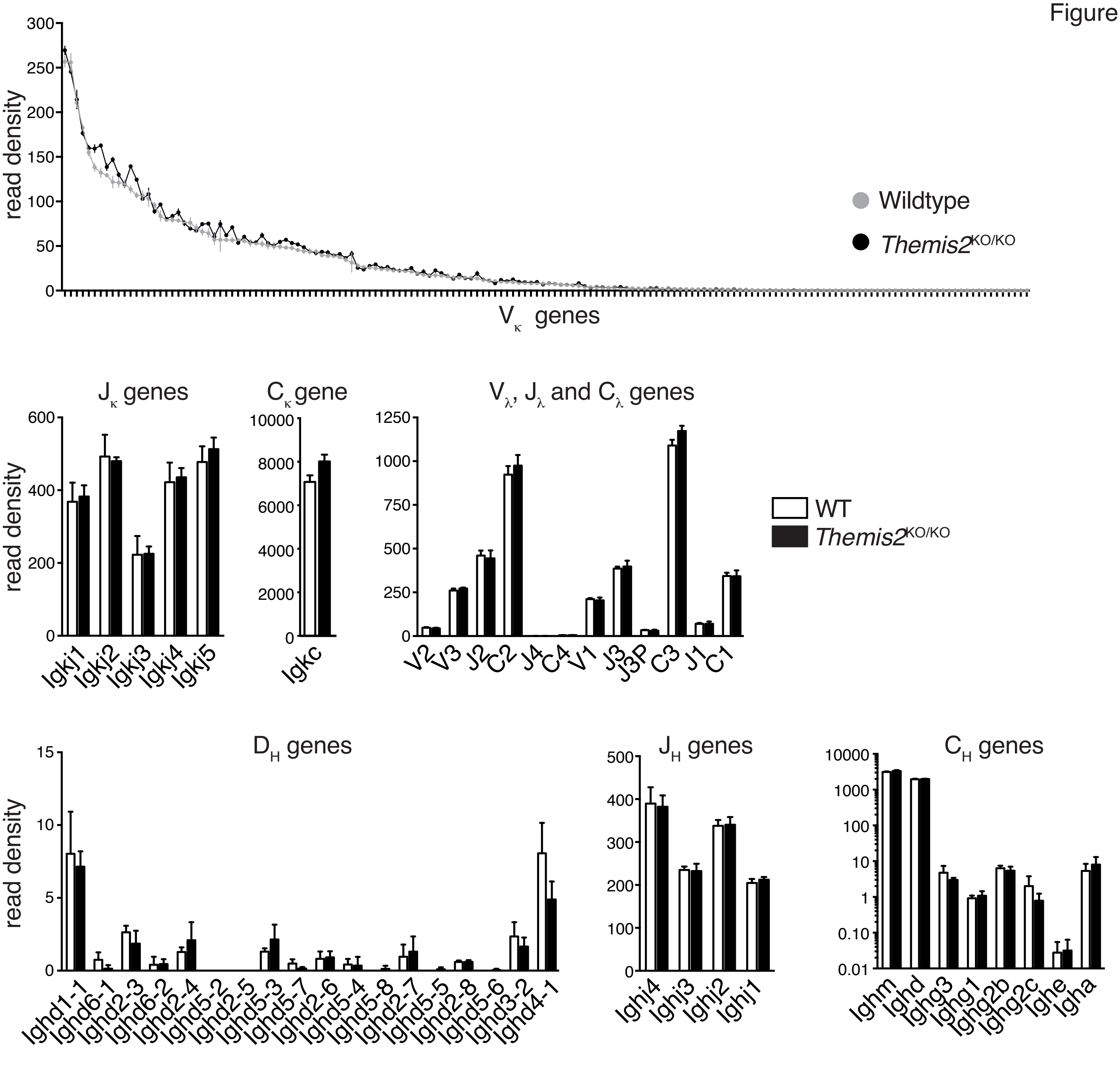

Supplemental Figure 3. Themis2-deficient follicular B cells show no change in usage of immunoglobulin genes. Expression of V_κ, J_κ, C_κ, V_λ , J_λ, C_λ, D_H, J_H and C_H genes in splenic B cells from WT or *Themis2$^{KO/KO}$* mice determined by RNAseq. V_κ genes are ordered by level expression in WT mice. Graphs show mean ± SEM of 3 biological replicates.
